# Small hepatic veins Budd–Chiari syndrome

**DOI:** 10.1007/s11239-013-0959-z

**Published:** 2013-06-28

**Authors:** Oliviero Riggio, Chiara Marzano, Alessia Papa, Chiara Pasquale, Maria Ludovica Gasperini, Antonietta Gigante, Dominique Charles Valla, Aurélie Plessier, Antonio Amoroso

**Affiliations:** 1 Gastroenterology, Department of Clinical Medicine, Sapienza University of Rome, Rome, Italy; 2 Department of Clinical Medicine, Sapienza University of Rome, Rome, Italy; 3 Service d’Hépatologie, Hôpital Beaujon, Clichy, France; 4 Internal Medicine, Department of Clinical Medicine, Sapienza University of Rome, Viale dell’Università 37, 00185 Rome, Italy

**Keywords:** Small hepatic veins, Budd–Chiari syndrome, Thrombosis

## Abstract

Budd–Chiari syndrome is a rare disorder characterized by hepatic venous outflow obstruction at any level from the small hepatic veins to the atrio-caval junction, in the absence of heart failure or constrictive pericarditis. Various imaging modalities are available for investigating the gross hepatic vascular anatomy but there are rare forms of this disease where the obstruction is limited to the small intrahepatic veins, with normal appearance of the large hepatic veins at imaging. In this cases only a liver biopsy can demonstrate the presence of a small vessels outflow block. We report two cases of small hepatic veins Budd–Chiari syndrome.

## Introduction

Budd–Chiari syndrome (BCS) is a rare disorder characterized by a hepatic venous outflow obstruction at any level from the small hepatic veins to the atrio-caval junction [1–3].

The major risk factors in primary BCS are inherited or acquired thrombophilic conditions. Other factors such as the use of oral contraceptives may also be involved [4].

The diagnosis of BCS should be suspected in case of abdominal pain, ascites, liver and spleen enlargement and portal hypertension, but is established only upon demonstration of an obstructed hepatic venous outflow tract. Diagnostic features are the demonstration of an altered blood flow in the hepatic veins, and/or obstructed hepatic veins or inferior vena cava, and/or intrahepatic or sub capsular hepatic venous collaterals. There are rare forms where the obstruction is limited to the small intrahepatic veins, with normal appearance of the large hepatic veins. In these case the diagnosis of BCS is not easy and the liver biopsy is the only way to demonstrate the presence of a small vessel outflow block.

We describe two cases of young patients with BCS due to the occlusion of the small hepatic veins.

## Case 1

A 28 years old woman was admitted to our Department because of abdominal distention and pain. The physical examination revealed hepatomegaly and ascites. Laboratory analyses revealed elevated total bilirubin of 2.45 mg/dL (normal 0.3–1.2 mg/dL), increased γ-glutamyltransferase (82 U/L; normal 5–36) and alkaline phosphatase (130 U/L: normal 35–104), INR was 1.45. The peripheral blood count was normal. All causes of liver disease (alcohol, drugs, viral, autoimmune, alfa1-antitrypsin deficiency, hemochromatosis, Wilson disease, celiac disease, NASH/NAFLD) were ruled out. Approximately about 5,000 mL of ascites were drained. Cytology was negative, the serum—ascites albumin gradient was 2.4 g/dL. The neutrophil cell count excluded an infection of the ascitic fluid.

**Fig. 1:**
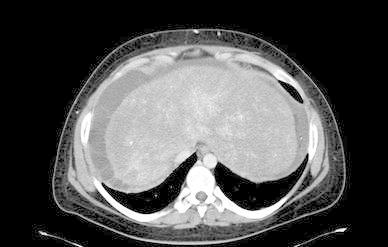
Abdominal CT scan showing inhomogeneous liver parenchymal enhancement after i.v. contrast injection, patency of the inferior vena cava with lack of visualization of the hepatic veins

Abdominal CT scan showed hepatomegaly, inhomogeneous liver parenchymal enhancement after i.v. contrast injection, enlargement of the caudate lobe (Fig. 1). At doppler examination the hepatic veins appeared to be patent and in all of them a flow was detected. Upper digestive endoscopy was negative for oesophageal varices.

**Fig. 2:**
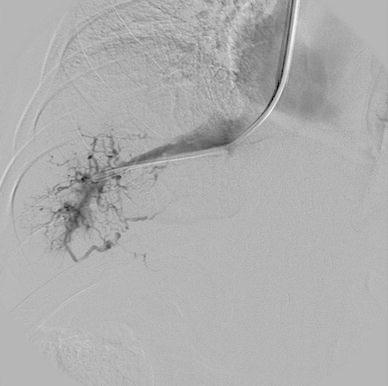
Venography showing a patent right hepatic vein with evidence of a spider web (tiny venous channels opacified from the catheter tip in the wedge position)

**Fig. 3:**
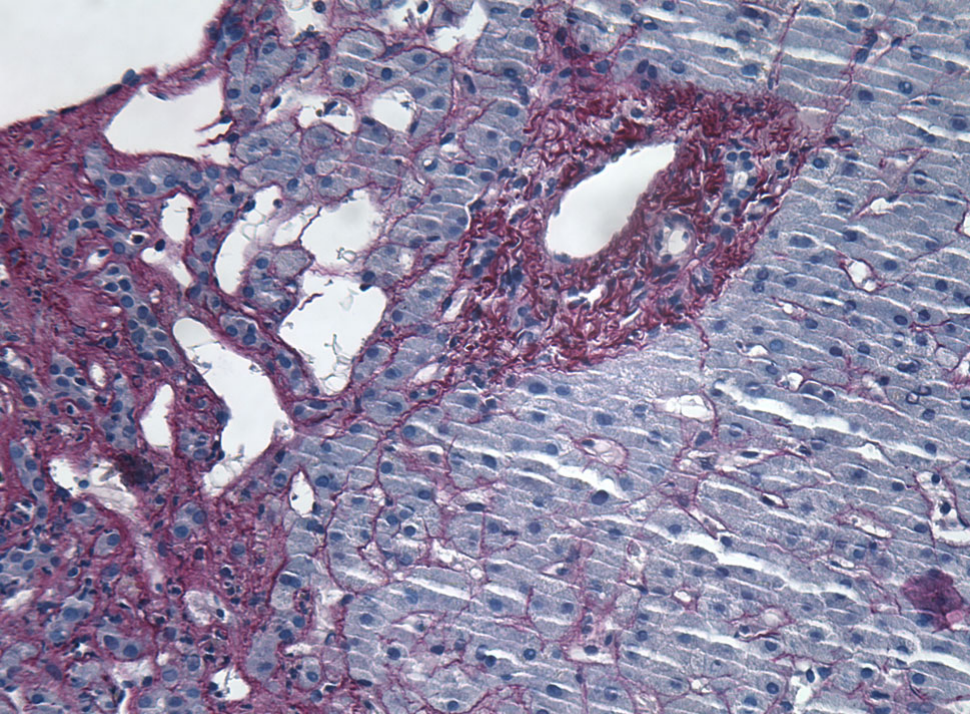
Needle biopsy Hematoxylin/Eosin ×100: there are marked dilated sinusoids and fibrosis of the perisinusoidal stroma

An hepatic vein venography confirmed that the hepatic veins were patent but a spider web (tiny venous channels opacified from the catheter tip in the wedge position) was evident (Fig. 2). A transjugular liver biopsy was therefore performed. Histological findings resulted compatible with the diagnosis of BCS, showing dilatation of the sinusoids, mild and focal perivenular inflammatory infiltrate and perivenular fibrosis (Fig. 3).

An extensive screening for hereditary thrombophilia (factor V Leiden mutation, prothrombin gene mutation, and inherited deficiencies for protein C, protein S and antithrombin) and acquired pro-thrombotic disease (myelo-proliferative disorders, anticardiolipin antibodies, lupus anticoagulant antibodies, paroxysmal nocturnal hemoglobinuria) was carried on. Myeloproliferative neoplasms were screened by the detection of Janus tyrosine kinase-2 gene (JAK-2 V617F) mutation. All these tests were negative but the value of Protein C was at 44 % of the normal range. Ascites was successfully treated with diuretics and the patient was discharged with oral anticoagulant therapy. Ten months later, despite diuretic therapy and oral anticoagulation, ascites recurred, as a worsening of liver function. Upper endoscopy revealed the presence of oesophageal varices. Percutaneous liver biopsy was carried on after withdrawal of oral anticoagulation. Histology showed a marked dilatation and congestion of the sinusoids, perivenular fibrosis and, at variance with the first biopsy, the presence of pseudo-lobules. The patient was submitted to TIPS [5] using a PTFE-covered-stent. The HVPG decreased from 18 mmHg to 5 mmHg. Ascites disappeared after TIPS and upper endoscopy performed 1 month after the procedure showed the regression of esophageal varices.

## Case 2

A 14 year-old boy was referred to our department because of low platelets count, mild hepatomegaly, splenomegaly and ascites detectable only at ultrasound. Since the age of 7 years, he had repeated episodes of haematuria, with negative urologic examinations, and recurrent episodes of ecchymoses after small traumas or even spontaneously. The patients was submitted to bone marrow biopsy, which excluded primary haematological disorders. Blood tests revealed ALT 54 U/L (n.v. <40) and AST 53 U/L (n.v. <40 U/L). All causes of liver disease (alcohol, drugs, viral, autoimmune, alfa1-antitrypsin deficiency, hemochromatosis, Wilson disease, celiac disease, NASH/NAFLD) were ruled out. Abdominal RM and CT scan showed asymmetrical hepatomegaly, inhomogeneous liver parenchymal enhancement after i.v. contrast injection, subcapsular hepatic venous collaterals, enlargement of the caudate lobe. The hepatic veins were patent and a flow was detectable at Doppler ultrasound. A liver biopsy showed sinusoidal congestion and atrophy of the hepatocytes lamina especially in the peri-venous area. Thus, a diagnosis of BCS due to the obstruction of the small hepatic veins was made. Endoscopy was negative for varices. The thrombophilic screening (factor V Leiden mutation, prothrombin gene mutation, inherited deficiencies for protein C, protein S and antithrombin, anticardiolipin antibodies, lupus anticoagulant antibodies, paroxysmal nocturnal hemoglobinuria), including V617F JAK2 mutation, was negative. Because of recurrent bleeding anticoagulation was not started. The patient is regularly followed-up as outpatient since 6 years. No deterioration of liver function neither the development of portal hypertension was observed.

## Discussion

The diagnosis of BCS is based on clinical and radiological findings. The site of obstruction may be determined through a non-invasive imaging as Doppler ultrasound, CT-scan, magnetic resonance or by venography. The main features are absence of flow in the hepatic veins, the presence of veno-venous collaterals and parenchymal alterations, such as signs of cirrhosis and nodulation. The gold standard for the diagnosis of BCS is hepatic veins and inferior vena cava angiography, which can show the presence of an obstacle in the hepatic veins and gross hepatic collaterals or a spider web. BCS is classified according to the location of the obstruction: small hepatic veins, large hepatic veins, inferior vena cava and combined obstruction of large hepatic veins and inferior vena cava [6]. So, the patency of the major hepatic veins does not exclude the presence of BCS. In fact, in small hepatic veins obstruction, the diagnosis is based on histological features only, and the diagnostic suspicion arises from clinical findings (ascites, hepato-splenomegaly, abdominal pain).

BCS due to the occlusion of small hepatic veins is a very rare clinical entity. It has been more often described associated with primary or secondary antiphospholipid syndrome (APS) [7–9] or paroxysmal nocturnal hemoglobinuria [10], where the presence of a thrombus in the small intrahepatic vessels can be observed. In cases not associated to APS, biopsy usually brings not specific evidence for an impaired blood outflow, including congestion, coagulative necrosis or simple loss of hepatocytes without inflammatory infiltrates and or fibrosis, and all of these features are predominant in the centrilobular area. These histological findings are therefore unspecific, being observable in any outflow obstructions such as those secondary to a block of the large hepatic veins, or to cardiac or pericardial disease. Thus, a combination of histological findings and imaging studies is necessary for the diagnosis of this rare condition.

Another possible differential diagnosis is with the veno-occlusive disease. The term veno-occlusive disease of the liver refers to a form of toxic liver injury characterized clinically by the development of hepatomegaly, ascites, and jaundice [11], and histologically by diffuse damage in the centrilobular zone of the liver. The cardinal histological features of this injury are marked sinusoidal fibrosis, necrosis of pericentral hepatocytes, and narrowing and eventual fibrosis of central veins. A more appropriate name for this form of liver injury is Sinusoidal obstruction syndrome. Sinusoidal obstruction syndrome usually occurs after a bone marrow or solid organ transplantation as well as a consequence of drug toxicity, and is histologically characterized by the presence of endophlebitis leading to activation of the coagulation cascade, and clot formation, with fibrin-related plugs, intra-cellular fluid entrapment and cellular debris which progressively occlude sinusoids [11].

Independently from the site of obstruction, the patient may be treated, stepwise, with medical therapy, TIPS or liver transplantation [12]. The possibility of mechanical relief of hepatic outflow obstruction is of course not possible in these patients. Any underlining disease should be also treated. The problem is the indication for anticoagulation, for which there are not clearly stated guidelines in patients with BCS due to the obstruction of the small hepatic veins. Despite an extensive diagnostic workout, we were able to detect a protein C reduction only in one of our two patients. It should be noted that protein C deficiency is a difficult diagnosis in a patient with liver disease [2], as this protein is synthesized by the liver. As the levels of other proteins synthesized by the liver were normal the diagnosis of protein C deficiency was made and oral anticoagulation was started. In the second patient, any inherited or acquired thrombophilic disorder was excluded and anticoagulation therapy was not carried on, also on the basis of a positive history for repeated episodes of haematuria, and recurrent episodes of ecchymoses after small traumas or even spontaneously. We have no data to support these decisions and guidelines on the treatment of these cases are not available. Due to the few reports of small hepatic veins BCS the prognosis is also unknown.

In conclusion, we described two patients with a very rare condition. The suspicion should be based on the clinical picture, which is very similar to the presentation of classic BCS, but the diagnosis should be supported by histology. The indication to perform a liver biopsy is based of clinical symptoms and on the appearance of the liver parenchyma at the imaging technique. The demonstration of flow in the large hepatic veins should not exclude the disease.

## References

[1] Janssen HL, Garcia-Pagan JC, Elias E (2003). Budd–Chiari syndrome: a review by an expert panel. J Hepatol.

[2] Valla DC (2009). Primary Budd–Chiari syndrome. J Hepatol.

[3] Darwish-Murad S, Plessier A, Hernandez-Guerra M (2007). A prospective study on 163 patients with Budd–Chiari syndrome: results from the European Network for Vascular Disorders of the Liver (EN-VIE). J Hepatol.

[4] Horton JD San Miguel FL, Membreno F, et al. (2008) Budd–Chiari syndrome: illustrated review of current management. Liver Int 28(4):455–466 (Review. Erratum in. Liver Int 28(6):898) 10.1111/j.1478-3231.2008.01684.x 18339072

[5] Plessier A, Sibert A, Consigny Y (2006). Aiming at minimal invasiveness as a therapeutic strategy for Budd–Chiari syndrome. Hepatology.

[6] Ludwig J, Hashimoto E, McGill DB (1990). Classification of hepatic venous outflow obstruction: ambiguous terminology of the Budd–Chiari syndrome. Mayo Clin Proc.

[7] Nakamura H, Uehara H, Okada T (1989). Occlusion of small hepatic veins associated with systemic lupus erythematosus with the lupus anticoagulant and anti-cardiolipin antibody. Hepatogastroenterology.

[8] Uthman I, Khamashta M (2007). The abdominal manifestations of the antiphospholipid syndrome. Rheumatology.

[9] Velasco M, Chesta J, Grisanti M (1993). Post sinusoidal obstruction of the hepatic venous flow associated with antiphospholipid syndrome in 3 cases. Rev Med Chil.

[10] Valla D, Dhumeaux D, Babany G (1987). Hepatic vein thrombosis in paroxysmal nocturnal hemoglobinuria. A spectrum from asymptomatic occlusion of hepatic venules to fatal Budd–Chiari syndrome. Gastroenterology.

[11] Senzolo M, Riggio O, Primignani M. Vascular disorders of the liver: Recommendations from the Italian Association for the Study of the Liver (AISF) ad hoc committee. *Dig Liver Dis*. 2010 Dec 24 10.1016/j.dld.2010.11.006 21185794

[12] DeLeve LD, Valla DC (2009). Garcia-Tsao G; American Association for the Study Liver Diseases. Vascular disorders of the liver. Hepatology..

